# Maskless head and neck radiotherapy using surface‐guided radiation therapy: initial clinical feasibility of real‐time setup, intrafraction monitoring, and automatic beam‐hold

**DOI:** 10.1002/acm2.70809

**Published:** 2026-09-25

**Authors:** Adi Robinson, Shravan Kandula

**Affiliations:** ^1^ Department of Radiation Oncology Medical Physics AdventHealth Celebration Celebration Florida USA

**Keywords:** cone‐beam CT, head and neck radiotherapy, intrafraction motion, maskless immobilization, patient comfort, SGRT, surface‐guided radiation therapy

## Abstract

**Background:**

Thermoplastic immobilization remains standard for head and neck radiation therapy, but rigid face masks may produce clinically meaningful anxiety, claustrophobia, and treatment intolerance in some patients. Surface‐guided radiation therapy (SGRT) provides non‐ionizing surface setup guidance and continuous intrafraction motion monitoring, potentially enabling reduced‐immobilization treatment approaches.

**Purpose:**

To describe and initially evaluate the clinical feasibility of a thermoplastic‐maskless head and neck radiation therapy workflow using a posterior dorsal shell, SGRT‐guided setup, cone‐beam computed tomography (CBCT) verification, and real‐time intrafraction monitoring with automatic beam‐hold.

**Methods:**

Ten patients with head and neck cancer were treated without a thermoplastic face mask. Patients were positioned in a posterior dorsal shell. Daily setup began with SGRT followed by CBCT verification and couch correction. During delivery, SGRT (AlignRT v6.3, Vision RT, London, UK) monitored head position in six degrees of freedom (6DoF); automatic beam‐hold was triggered when translational displacement exceeded 2 mm or rotational displacement exceeded 2°. Post‐treatment CBCT and SGRT log files were reviewed for every fraction to characterize residual displacement, threshold events, and repositioning. Intrafraction motion and residual displacement were summarized as medians with interquartile range (IQR).

**Results:**

Across 330 delivered fractions, sgrt‐recorded intrafraction motion was small:: per‐axis signed real‐time deltas had a median [IQR] of 0.10 mm [−0.15, 0.30] translationally and 0.10° [−0.05, 0.20] rotationally. No fraction had a mean real‐time delta exceeding the ± 2 mm/ ± 2° tolerance, and automatic beam‐hold interruptions occurred in approximately 2% of fractions (7 of 330); no fraction was abandoned. Per‐axis post‐treatment CBCT residuals were −0.01 mm [−0.04, 0.05] translationally and 0.15° [−0.40, 0.55] rotationally. All patients completed the maskless course; 3 of 10 had previously declined or been unable to tolerate mask‐based treatment, and all reported the maskless workflow to be well tolerated on an end‐of‐course questionnaire.

**Conclusions:**

A thermoplastic‐maskless head and neck workflow using SGRT, CBCT verification, predefined beam‐hold thresholds, and dorsal shell support was feasible in this initial 10‐patient experience, maintaining small observed intrafraction motion while directly addressing mask intolerance. Larger prospective evaluation with validated patient‐reported outcomes, dosimetric analysis, and multi‐institutional QA is warranted.

## 1 INTRODUCTION

Rigid thermoplastic face masks are a routine component of definitive and adjuvant head and neck radiation therapy. Their purpose is straightforward: to improve reproducibility during simulation and daily treatment, constrain intrafraction motion, and support margin selection for highly conformal intensity‐modulated or volumetric modulated treatments. However, the same physical features that make a closed mask effective—facial contact, restriction, and fixation to the treatment couch—can be a barrier for patients with claustrophobia, pain, airway concerns, treatment‐related anatomical change, or severe anticipatory anxiety. Prospective and qualitative studies have shown that mask‐related distress is common enough to warrant systematic screening and mitigation rather than being treated as an isolated inconvenience.^1^, ^2^, ^3^

Open‐face masks were developed to reduce facial confinement while retaining a degree of rigid immobilization. In parallel, SGRT has matured from an adjunct setup technology into a workflow platform for patient positioning, surface‐agreement assessment, intrafraction monitoring, and beam gating. AAPM Task Group 302 describes SGRT as a technology that requires defined commissioning, periodic quality assurance, risk analysis, and clear clinical procedures when used for real‐time motion management.^4^ These recommendations complement earlier quality‐assurance guidance for nonradiographic localization systems.^5^

For head and neck treatments, SGRT introduces a different safety model. Rather than relying exclusively on passive immobilization, the treatment team uses an external optical surface as a real‐time surrogate for patient position. Published studies of SGRT in head and neck, cranial, and intracranial applications have generally shown improved or comparable setup accuracy, useful intrafraction monitoring, and improved comfort when open‐face systems are used.^6^, ^7^, ^8^, ^9^, ^10^, ^11^, ^12^ However, open‐face masks still impose a physical immobilization device over the head and face, and facial expression or mobile anatomy can create apparent SGRT positional errors if regions of interest are not selected carefully.^13^

This study describes an institutional maskless head and neck workflow in which the face mask is removed entirely. The patient is supported by a posterior dorsal shell, positioned with SGRT, verified with CBCT, and monitored continuously during delivery with automatic beam‐hold at predefined translational and rotational thresholds. The goal was not to demonstrate superiority over conventional immobilization, but to document a reproducible clinical implementation, initial geometric evaluation, workflow controls, and patient‐experience signals that may inform broader evaluation.

## 2 MATERIALS AND METHODS

### 2.1 Study design and oversight

**FIGURE 1:**
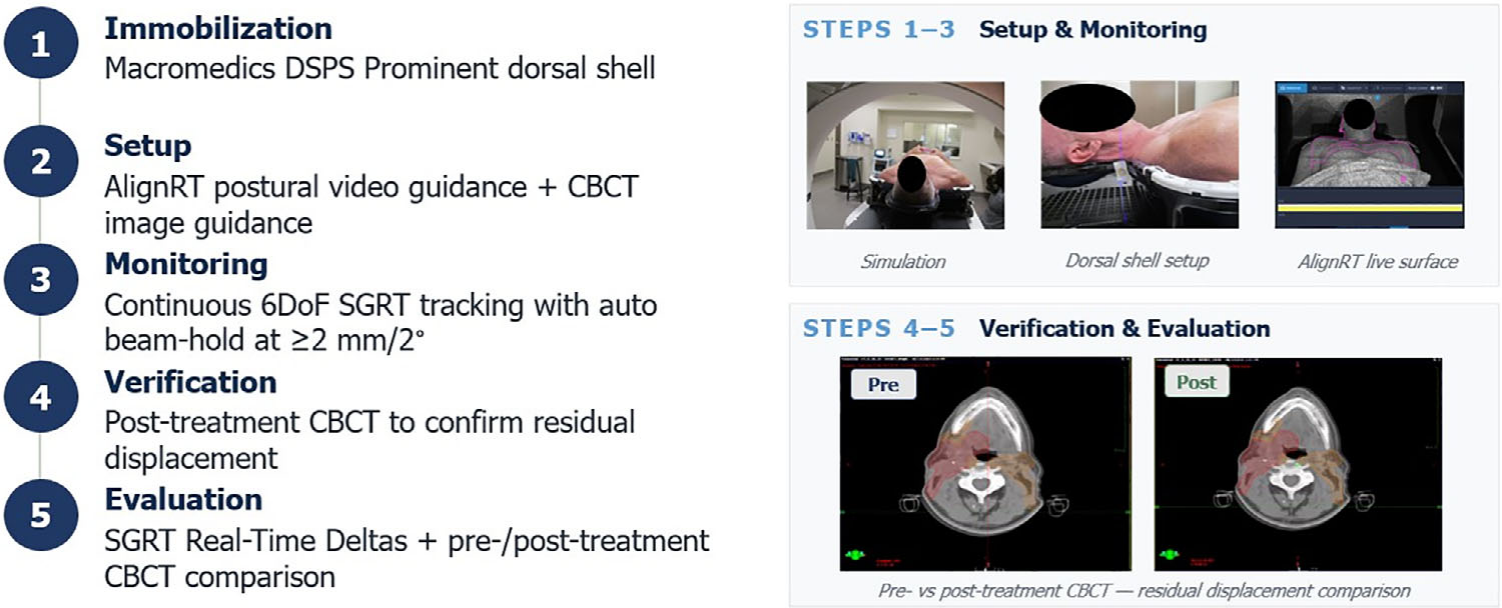
Maskless head and neck SGRT workflow. The five‐step workflow comprises (1) posterior dorsal shell immobilization without thermoplastic facial confinement; (2) SGRT postural setup combined with CBCT image guidance; (3) continuous 6DoF SGRT monitoring with automatic beam‐hold at ≥2 mm/≥2°; (4) post‐treatment CBCT verification of residual displacement; and (5) end‐of‐fraction evaluation using SGRT real‐time deltas and pre‐/post‐treatment CBCT comparison. Right panels: clinical photographs of CT simulation, dorsal shell setup, the AlignRT live surface, and representative pre‐ and post‐treatment CBCT images for residual‐displacement comparison.

This was a single‐institution technical implementation and initial clinical evaluation of a maskless SGRT workflow for selected head and neck patients. The reporting basis consisted of per‐fraction SGRT log review, pre‐ and post‐treatment CBCT registration, treatment‐event documentation, and an end‐of‐course patient questionnaire. This work was carried out as a departmental quality‐assurance/quality‐improvement project; under institutional policy, formal institutional review board approval was not required. Post‐treatment CBCT was acquired daily for every fraction as part of this evaluation; beyond this evaluation, post‐treatment CBCT is expected to be used periodically (e.g., weekly) to verify positioning. Outcomes were summarized descriptively because the cohort was intentionally limited to early clinical adoption and was not powered for formal statistical hypothesis testing. Figure 1 summarizes the workflow and verification sequence.

### 2.2 Patient eligibility and candidacy assessment

Patients were considered eligible when they presented with a head and neck indication suitable for conventional fractionation, could voluntarily maintain head position, were willing to attempt the maskless workflow, and had treating‐physician approval. Patients were not selected for maskless treatment when they had involuntary tremor, a movement disorder, severe cognitive impairment limiting cooperation, a preference for a conventional mask, or physician preference for the standard masked workflow. Final eligibility was determined collaboratively by the physician, patient, and physics team. Patient and treatment characteristics for the cohort are summarized in Table 1.

**TABLE 1:** Patient and treatment characteristics for the maskless head and neck SGRT cohort (*n* = 10).

Characteristic	Cohort (*n* = 10)
Age, years—median	63
Sex—*n* male / *n* female	10 male / 0 female
Primary treatment site	Larynx and/or base of tongue
Prescription dose	66 Gy
Number of fractions	33
Planning technique	VMAT, 2–3 arcs
CTV‐to‐PTV margin	3 mm
Approximate treatment (beam‐on) time	6–8 min
Bolus used	None (0 of 10)

At CT simulation, each patient's ability to hold a stable, reproducible head position without a mask was assessed before committing to the maskless workflow. With the patient in the treatment position, the simulation therapist, the treating physician, and the physicist observed the patient for 1–2 min to confirm that the patient could remain still; SGRT tolerances cannot be verified at simulation, so this assessment was observational. Because the treatment system automatically holds the beam for any deviation beyond tolerance, and because we sought to evaluate the workflow across a range of scenarios, patients were excluded only if they could not remain still during this evaluation period.

### 2.3 Simulation and immobilization

Each patient was simulated and treated using a Macromedics DSPS Prominent posterior dorsal shell (Macromedics, Roden, Netherlands) without a thermoplastic facial mask. Patients were treated on a Varian TrueBeam linear accelerator (Varian Medical Systems, Palo Alto, CA, USA). AlignRT (v6.3; Vision RT, London, UK) was used for surface guidance throughout; the manufacturer‐stated positioning accuracy of the system is submillimeter. The dorsal shell provided posterior head, neck, and upper‐shoulder support but did not impose anterior facial confinement. Standard CT simulation, target delineation, treatment planning, image guidance, and physician review were otherwise performed according to institutional head and neck practice.

The SGRT reference surface used for daily positioning was the external body (skin) contour derived from the planning CT dataset. No patient in this cohort required bolus. When bolus is required in a maskless setup, it can be molded and taped to the patient—as is done for other maskless anatomical sites treated with bolus—to keep it in place during treatment; bolus handling in the maskless workflow warrants dedicated evaluation.

### 2.4 SGRT region‐of‐interest strategy

**FIGURE 2:**
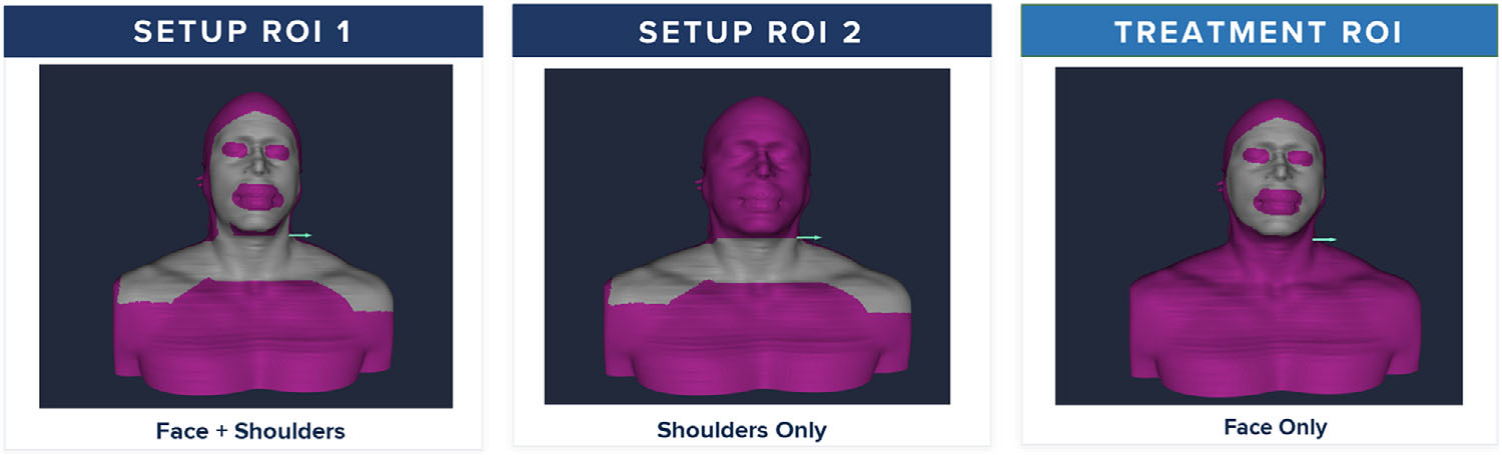
AlignRT region‐of‐interest (ROI) configuration for maskless head and neck SGRT. Setup ROI 1 (face + shoulders) is used for initial coarse alignment; Setup ROI 2 (shoulders only) refines torso and shoulder posture independent of head rotation; the Treatment ROI (face only) is used for intrafraction beam‐hold monitoring during delivery. Magenta regions indicate surface area included in registration. Stable surfaces (forehead, nasal bridge, cheeks, upper chest) are included; mobile surfaces (mandible, lips, neck folds) are excluded.

A staged region‐of‐interest (ROI) strategy was used because the head and shoulders can rotate about different axes. Setup ROI 1 included the face and shoulders and was used for initial gross alignment. Setup ROI 2 included the shoulders only and was used to refine torso and shoulder posture independent of head rotation. The treatment ROI included the face only and was used for intrafraction monitoring during beam delivery. Stable surfaces such as the forehead, bridge of the nose, cheeks, and upper chest were favored. Mobile structures, including the mandible, lips, and neck folds, were excluded whenever possible. Before delivery, the SGRT deformation view and color‐map agreement were reviewed to assess non‐rigid disagreement and surface quality. Figure 2 shows the ROI configurations.

This ROI approach was designed to reduce two failure modes: accepting an incorrect shoulder posture because the face appeared aligned, and triggering unnecessary beam‐holds because mobile facial or neck surfaces were included in the monitoring ROI. Published work on open‐face SGRT has shown that facial expression and ROI choice can affect apparent positional corrections, supporting the need for explicit ROI templating and staff training.^13^

### 2.5 Daily treatment workflow

For each fraction, therapists positioned the patient using the SGRT surface with Setup ROI 1 followed by Setup ROI 2. A pre‐treatment CBCT was then acquired, registered, and applied for treatment‐position correction, and a new SGRT reference surface was captured at the corrected treatment position for intrafraction monitoring. During delivery, SGRT monitored translational and rotational motion in six degrees of freedom. The beam‐hold threshold was set to 2 mm translational displacement or 2° rotational displacement; when the threshold was crossed, delivery was interrupted automatically. System latency was not measured as part of this study; it is assessed during annual quality assurance for this system and is on the order of 135 ms.

The response to a beam‐hold followed a predefined, documented sequence. The patient was first coached to return to the reference position. If the surface returned to within tolerance through coaching alone, delivery resumed without additional imaging. If the displacement persisted, or if the posture was judged non‐reproducible, treatment was paused and the patient was repositioned; a repeat CBCT was then acquired to re‐verify internal anatomy before delivery resumed. In other words, repositioning using SGRT alone was used only for transient, self‐correcting surface excursions, whereas a repeat CBCT was required whenever the patient was physically repositioned or when reproducibility was in question.

A post‐treatment CBCT was acquired to estimate residual displacement between the pre‐treatment (corrected) treatment position and the end‐of‐treatment position. SGRT real‐time deltas (RTDs) were reviewed per fraction to characterize threshold events, mean and median intrafraction motion, and the need for repositioning. Table 2 summarizes the daily treatment workflow and the technical control associated with each step.

**TABLE 2:** Maskless SGRT treatment workflow and associated technical controls.

Stage	Technical action	Primary control
Simulation	Dorsal shell setup without face mask; CT simulation; reference (external body) surface derived from the planning CT.	Reproducible posterior support while avoiding facial confinement.
Initial treatment setup	SGRT surface with live deltas using Setup ROI 1, then Setup ROI 2.	Separates gross head/shoulder posture from shoulder refinement.
Image guidance	Pre‐treatment CBCT registration and couch correction according to institutional head and neck protocol.	CBCT remains the internal‐anatomy reference before treatment delivery.
Treatment monitoring	Continuous 6DoF SGRT with automatic beam‐hold at ≥2 mm translation or ≥2° rotation.	Continuous surface monitoring provides an active positional check in place of rigid anterior facial fixation.
Action upon beam‐hold	Pause and coach the patient; allow self‐correction when the surface returns to tolerance; reposition and re‐image (repeat CBCT) if displacement persists or posture is non‐reproducible.	Standardizes staff action upon a beam interruption and avoids ad hoc tolerance decisions.
Post‐treatment verification	Post‐treatment CBCT and SGRT log review.	Quantifies residual displacement and supports QA of threshold events.

### 2.6 Outcome definitions and statistical analysis

The primary technical outcomes were SGRT‐recorded intrafraction translational and rotational motion, post‐treatment CBCT residual displacement, the frequency of fractions with at least one SGRT threshold violation, and the frequency of fractions requiring repositioning. Intrafraction motion was computed as the per‐fraction mean of the 6DoF RTD magnitude, sampled continuously at 15–25 frames per second across each beam‐delivery interval; per‐axis values (vertical, longitudinal, lateral, pitch, roll, yaw) and a vector‐magnitude summary are reported. Post‐treatment CBCT residual displacement was defined as the rigid‐registration shift between the corrected pre‐treatment position and the post‐treatment CBCT acquired on the same fraction. A threshold violation was defined as any beam‐hold event caused by displacement exceeding the 2 mm or 2° tolerance. Repositioning was defined as therapist intervention beyond simple coaching or self‐correction, as described in Section 2.5.

Continuous outcomes (intrafraction RTDs and post‐treatment CBCT residuals) are summarized as per‐axis signed medians with interquartile range (IQR). Because real‐time delta distributions are bounded and typically skewed rather than normally distributed, and because the raw per‐sample RTDs recorded during monitoring were reported directly, medians and IQRs were used rather than assuming normality; a formal normality test was not performed. The frequency of automatic beam‐hold interruptions was recorded. Population‐based CTV‐to‐PTV margins were estimated from the observed per‐axis intrafraction motion using the van Herk formulation (2.5Σ + 0.7σ) and compared with the 3 mm margin used clinically; because per‐patient decomposition into systematic and random components was not available, the systematic and random terms were approximated from the pooled per‐axis distribution and the resulting estimate is therefore approximate.

## 3 RESULTS

### 3.1 Cohort and treatment delivery

Ten patients were treated with the maskless SGRT workflow for a total of 330 delivered fractions. No fraction was abandoned because of inability to tolerate the maskless setup or because of motion‐management failure. Patient demographics, primary treatment site, fractionation, dose, planning technique, and margins are summarized in Table 1.

### 3.2 Geometric and workflow performance

**FIGURE 3:**
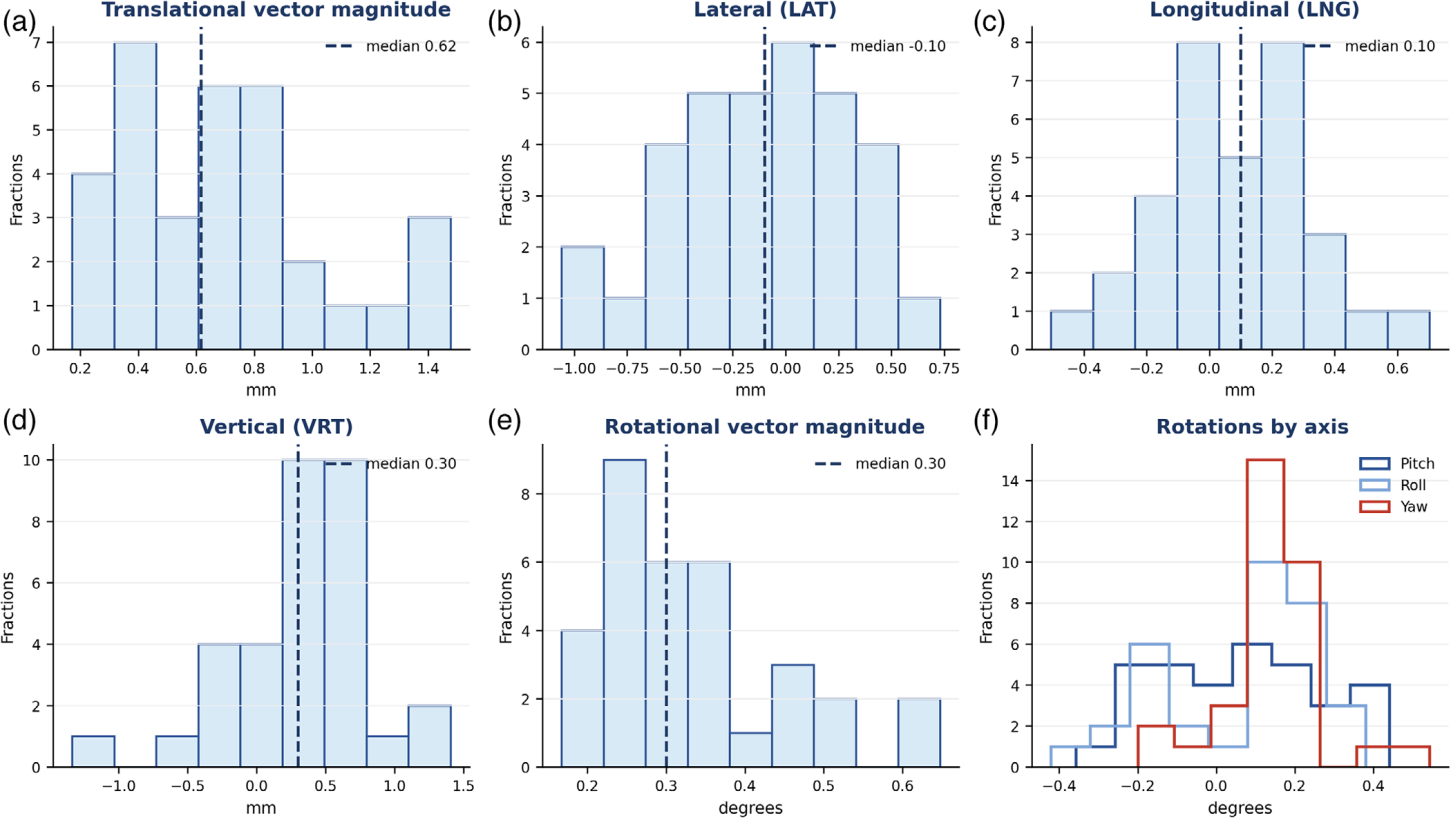
Distribution of per‐fraction mean SGRT intrafraction real‐time deltas (RTDs) across the cohort (33 fractions). Histograms show (a) translational vector magnitude, (b–d) the signed lateral, longitudinal, and vertical components, (e) rotational vector magnitude, and (f) the individual rotational axes; dashed lines mark the median. Per‐fraction mean displacements clustered near zero and remained within the ± 2 mm/ ± 2° tolerance. Automatic beam‐hold interruptions occurred in 7 of 330 fractions (2%).

SGRT‐recorded intrafraction motion was small across the cohort. Per‐axis signed real‐time deltas had a median [IQR] of 0.10 mm [−0.15, 0.30] translationally and 0.10° [−0.05, 0.20] rotationally; these outcomes are summarized in Table 3, and the per‐axis distributions are shown in Figure 3. Mean SGRT real‐time deltas remained within the ± 2 mm/ ± 2° envelope for all 330 fractions, and no fraction had a mean real‐time delta that exceeded tolerance. Automatic beam‐hold interruptions, triggered by transient surface excursions beyond tolerance, occurred in approximately 2% of fractions (7 of 330); in these events the beam held automatically and the patient was coached back to tolerance before delivery resumed; when a beam‐hold could not be resolved by coaching alone, the patient was repositioned and a repeat CBCT was acquired before resuming (Section 2.5).

**TABLE 3:** Initial clinical and geometric outcomes for the maskless head and neck SGRT cohort (*n* = 10; 330 fractions).

Outcome	Value (*n* = 10; 330 fractions)	Interpretive note
Patients treated	10	Selected head and neck patients approved for the maskless workflow.
Fractions delivered	330	All fractions completed; no fraction abandoned.
Intrafraction translation, SGRT—per‐axis median [IQR]	0.10 [−0.15, 0.30] mm	Signed per‐axis real‐time deltas; distribution shown in Figure 3.
Intrafraction rotation, SGRT—per‐axis median [IQR]	0.10 [−0.05, 0.20]°	
Post‐treatment CBCT residual—median [IQR], translational	−0.01 [−0.04, 0.05] mm	Signed per‐axis residual between corrected and end‐of‐treatment position.
Post‐treatment CBCT residual—median [IQR], rotational	0.15 [−0.40, 0.55]°	
Fractions with mean RTD exceeding tolerance	0 of 330	Mean SGRT deltas remained inside the ± 2 mm/ ± 2° envelope.
Automatic beam‐hold interruptions	7 of 330 (2%)	Triggered by transient surface excursions beyond tolerance; patients self‐corrected with coaching.
Estimated CTV‐to‐PTV margin (van Herk, translational)	≈1.4 mm	Approximate (SD ≈ 0.44 mm); compared with the 3 mm margin used clinically.

*Note*: Intrafraction and residual values are per‐axis signed medians with interquartile range; the van Herk margin is an approximate estimate.

Post‐treatment CBCT residual displacement was likewise small, with per‐axis medians [IQR] of −0.01 mm [−0.04, 0.05] translationally and 0.15° [−0.40, 0.55] rotationally. Using the observed per‐axis intrafraction motion (translational standard deviation ≈0.44 mm) and the conservative assumption that the systematic and random components each approximate this value, a van Herk margin estimate (2.5Σ + 0.7σ) yields approximately 1.4 mm, well within the 3 mm CTV‐to‐PTV margin used clinically, which additionally accommodates setup, delineation, and penumbral uncertainties. A definitive margin calculation would require decomposition into per‐patient systematic and random components.

### 3.3 Patient experience

Patient experience was assessed with a brief, investigator‐designed questionnaire administered at the end of treatment. Patients were asked how they tolerated treatment and how their experience changed over the course of care—from the first fraction, when anticipatory anxiety was typically highest, to the end of treatment. They were also shown both full‐face and open‐face thermoplastic masks and asked how they would feel about being treated in each.

All 10 patients completed the prescribed course without conversion to a masked workflow. Three of the 10 had declined, or reported that they could not tolerate, mask‐based treatment before starting, and for these patients the maskless workflow enabled treatment that might otherwise have required sedation or alternative management. Patients generally reported that treatment became easier to tolerate as the course progressed, and, when shown the full‐face and open‐face masks, indicated that they preferred the maskless experience. No validated patient‐reported‐outcome or anxiety instrument was used in this initial implementation; incorporating one is a priority for prospective evaluation.

## 4 DISCUSSION

This study describes the feasibility of treating selected head and neck patients without a thermoplastic face mask using a workflow that combines dorsal shell support, SGRT setup, CBCT verification, continuous SGRT monitoring, and a predefined beam‐hold response. In 330 fractions, mean SGRT‐recorded intrafraction motion remained small, threshold‐triggered intervention was infrequent, and no treatment fraction was abandoned. These findings support the central premise that maskless head and neck treatment is not simply removal of an immobilization device; it is a deliberately engineered motion‐management workflow.

The workflow aligns with the broader evolution of SGRT. SGRT is most clinically valuable when it is implemented as a process with commissioning, tolerance selection, staff training, failure‐mode analysis, and routine QA rather than as a passive camera overlay. In this implementation, CBCT was retained as the pre‐treatment internal‐anatomy verification step, while SGRT provided continuous temporal information that CBCT alone cannot. This complementarity is consistent with published clinical experience in head and neck and other cranial applications, where surface monitoring can detect transient deviations that may not be captured by pre‐ and post‐treatment imaging alone. SGRT nevertheless remains a surface surrogate and is not a replacement for internal‐anatomy verification in this workflow.

The clinical rationale is also patient‐centered. Mask‐related anxiety has been reported prospectively and qualitatively, and patient narratives frequently emphasize the moment of immobilization and fixation to the couch as a trigger.^2^, ^3^ For some patients, mitigation strategies such as coaching, music, anxiolytics, or reduced‐immobilization masks may be adequate; for others, the mask itself becomes the limiting factor for treatment acceptance. In the present cohort, 3 of 10 patients had declined or could not tolerate mask‐based treatment, making the maskless option a potential treatment‐enabling intervention rather than a comfort measure alone.

The results should be interpreted in the context of recent maskless and open‐face literature. Essers and colleagues^14^ reported a maskless head and neck workflow with SGRT and CBCT, showing acceptable intrafraction margins when motion greater than 2 mm was corrected. A subsequent clinical cohort using individual dorsal shells and SGRT—the most directly analogous published series—reported treatment interruptions in 3.5% of fractions using 2 mm/2° thresholds and supported a 3 mm total CTV‐to‐PTV margin for the maskless approach.^15^ The current institutional experience is directionally consistent with those reports: our 2% beam‐hold interruption rate (7 of 330 fractions) is comparable to their 3.5% interruption rate, although differences in cohort selection, fraction counts per patient, treatment technique, and beam‐hold response criteria limit direct comparison. The OPEN phase III trial, comparing open‐face to closed masks, similarly demonstrated that surface monitoring detects transient deviations that may not be captured by CBCT alone, further supporting the role of SGRT as an active safety component in reduced‐immobilization workflows.^12^

### 4.1 Technical considerations for implementation

Three implementation details were essential. First, patient selection was conservative: patients had to be able to cooperate, voluntarily maintain head position, understand the beam‐hold response, and tolerate coaching, and candidacy was assessed at simulation (Section 2.2). Second, ROI selection was standardized; the treatment ROI intentionally excluded the mandible, lips, and neck folds because these surfaces can move independently of bony anatomy and produce apparent SGRT shifts. A natural refinement, suggested by hybrid open‐face designs and by the OPEN trial experience,^12^ is to further subdivide the monitoring ROI into independent facial and clavicular/upper‐chest regions so that head and shoulder motion can be tracked separately during delivery; this is a candidate for prospective evaluation. Third, therapists used a consistent, documented response to threshold events: beam‐hold was treated as a safety feature, not a failure, and patients were coached to self‐correct before more disruptive repositioning was undertaken.

### 4.2 Commissioning and quality‐assurance considerations

Safe adoption of a maskless SGRT workflow depends on several program‐level controls that follow directly from TG‐302.^4^ Before clinical use, the institution should confirm dorsal‐shell fit and reproducible neutral head/shoulder posture at simulation, and validate the SGRT reference surface against the planning CT external contour. A standardized ROI template (Setup ROI 1, Setup ROI 2, and a face‐only treatment ROI) should be defined, with mobile anatomy excluded and deformation/color‐map review required before delivery. The 2 mm/2° beam‐hold tolerance should be validated against the institution's image‐guidance and margin policy. Pre‐treatment CBCT should be retained as the internal‐anatomy reference, with an explicit rule for when a repeat CBCT is required after repositioning (Section 2.5). Finally, a written therapist script for the beam‐hold response, together with periodic review of SGRT logs, threshold‐event frequency, repositioning frequency, and post‐treatment CBCT residuals, helps ensure that staff actions upon a beam interruption remain consistent and documented during early implementation. Structured collection of patient‐experience measures is recommended so that the primary motivation for the workflow—patient comfort—is tracked as rigorously as its geometric performance.

### 4.3 Limitations

This report has several limitations. The cohort was small and intentionally selected, so the results should not be generalized to all head and neck patients. The evaluation was single‐institution and descriptive, and was not designed as a comparative study; patients did not undergo mask‐based treatment, so the questionnaire reflects acceptability of the maskless workflow rather than a direct preference between masked and maskless treatment, and no validated patient‐reported‐outcome or anxiety instrument was used. Post‐treatment CBCT residual displacement characterizes end‐of‐fraction position but is not equivalent to continuous internal‐target tracking. Bolus was not required in this cohort, and bolus handling in a maskless setup will require dedicated evaluation. Finally, dosimetric consequences of the observed intrafraction motion were estimated only through margin calculation and were not assessed by delivered‐dose reconstruction.

Future work should include prospective, multi‐institutional enrollment with standardized inclusion criteria, validated patient‐reported outcomes, predefined SGRT/CBCT analysis methods, correlation of SGRT and CBCT motion estimates, dosimetric assessment of intrafraction motion, and formal failure‐mode‐and‐effects analysis. A supplementary technical appendix providing ROI templates, therapist scripts, and log‐review procedures may also be useful for centers adopting this workflow.

## 5 CONCLUSIONS

A maskless head and neck radiotherapy workflow using dorsal shell support, SGRT‐guided setup, CBCT verification, and continuous SGRT beam‐hold monitoring was feasible in an initial cohort of 10 selected patients and 330 fractions. The workflow maintained small observed intrafraction motion, triggered automatic beam‐hold interruptions in only 2% of fractions (7 of 330), and was well tolerated and reported as acceptable by all treated patients. For selected patients with mask anxiety or intolerance, maskless SGRT may function as both a technical motion‐management strategy and a treatment‐enabling patient‐comfort intervention. Broader adoption should proceed with conservative patient selection, explicit ROI templates, CBCT validation, documented threshold‐response procedures, and prospective outcome collection.

## AUTHOR CONTRIBUTIONS

Adi Robinson contributed to the conception and design of the study, clinical workflow development, data collection, data analysis and interpretation, manuscript drafting, critical revision, and final approval of the submitted manuscript. Shravan Kandula contributed to clinical implementation, patient evaluation, interpretation of clinical findings, critical manuscript revision, and final approval of the submitted manuscript.

## FUNDING

No study‐specific external funding was received. Data was generated as part of clinical implementation and routine departmental quality review.

## CONFLICT OF INTEREST STATEMENT

AdventHealth Celebration maintains a Center of Excellence agreement with Vision RT. AdventHealth Parker maintains a Professional Services Agreement with Vision RT. The authors report no other competing interests, and state that the clinical implementation and evaluation described in this manuscript reflect independent clinical experience and were not influenced by financial incentives.

## ETHICAL APPROVAL

This work was carried out as a departmental quality‐assurance/quality‐improvement project. Under institutional policy, formal institutional review board approval and separate written research consent were not required; patients provided standard clinical consent for treatment.

## Data Availability

Deidentified aggregate data supporting this technical note may be available from the corresponding author upon reasonable request and subject to institutional approval. Patient‐level imaging and SGRT log data are not publicly available as they may contain protected health information.
